# Gelatin Microsphere for Cartilage Tissue Engineering: Current and Future Strategies

**DOI:** 10.3390/polym12102404

**Published:** 2020-10-19

**Authors:** Shamsul Bin Sulaiman, Ruszymah Binti Haji Idrus, Ng Min Hwei

**Affiliations:** 1 Centre for Tissue Engineering and Regenerative Medicine, Faculty of Medicine, Universiti Kebangsaan Malaysia, Clinical Block, Jalan Yaacob Latiff, Cheras, Kuala Lumpur 56000, Malaysia; sshamsul@ppukm.ukm.edu.my (S.B.S.); ruszyidrus@gmail.com (R.B.H.I.); 2 Department of Physiology, Faculty of Medicine, Universiti Kebangsaan Malaysia, Kuala Lumpur 56000, Malaysia

**Keywords:** gelatin microsphere, microcarrier, cartilage, osteoarthritis, tissue engineering

## Abstract

The gelatin microsphere (GM) provides an attractive option for tissue engineering due to its versatility, as reported by various studies. This review presents the history, characteristics of, and the multiple approaches to, the production of GM, and in particular, the water in oil emulsification technique. Thereafter, the application of GM as a drug delivery system for cartilage diseases is introduced. The review then focusses on the emerging application of GM as a carrier for cells and biologics, and biologics delivery within a cartilage construct. The influence of GM on chondrocytes in terms of promoting chondrocyte proliferation and chondrogenic differentiation is highlighted. Furthermore, GM seeded with cells has been shown to have a high tendency to form aggregates; hence the concept of using GM seeded with cells as the building block for the formation of a complex tissue construct. Despite the advancement in GM research, some issues must still be addressed, particularly the improvement of GM’s ability to home to defect sites. As such, the strategy of intraarticular injection of GM seeded with antibody-coated cells is proposed. By addressing this in future studies, a better-targeted delivery system, that would result in more effective intervention, can be achieved.

## 1. Introduction

Cartilage diseases resulting from injury, trauma, or inflammatory diseases, such as osteoarthritis, are common orthopaedic problems that require definitive intervention [1]. Failure in conventional treatment has led researchers to find alternatives, of which tissue engineering provides an interesting option. Tissue engineering is a field of science that explores the production of engineered tissues while venturing into the body’s regenerative capabilities. Over the last few decades, researchers have explored the potential of tissue engineering in treating cartilage diseases; however, this approach is as yet under development.

Tissue engineering combines cells, scaffolds, and biologics to produce engineered tissue [2]. There is considerable literature on tissue engineering applications in the treatment of cartilage diseases [3]. Those that involve sophisticated scaffolds would require implanting the engineered tissue to the injured site of the cartilage via surgery [4]. Although several in vivo studies have reported disease improvement and tissue regeneration, in the context of clinical applications, the usage is still limited owing to the lack of tissue integration and surgical morbidity [4,5].

A more appealing approach to overcome the limitation of surgically implanted tissue involves utilizing a minimally invasive technique through intra-articular administration of microspheres. Microspheres are widely known for their usage in the in vitro expansion of cells inside a bioreactor, and as an efficient delivery system that periodically release drugs or biological agents. In tissue engineering, microspheres are utilized as a carrier for cells and as scaffoldings [6]. Currently, there are many commercially available microspheres, such as Cytodex 1, CultiSpher S and SphereCol [7]. In addition, some microspheres are made up of natural biomaterials, such as alginate, chitosan, silk, and gelatin [8,9,10,11].

**Figure 1:**
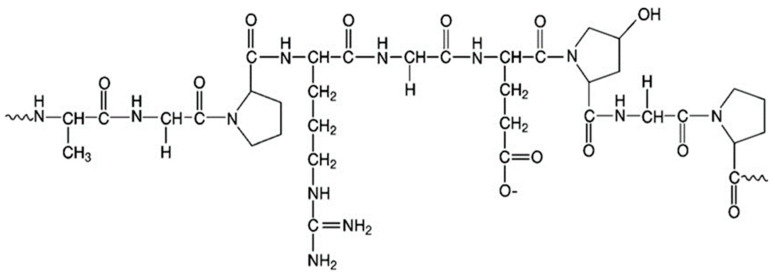
Basic chemical structure of gelatin.

Microspheres derived from biopolymers (polysaccharide- or protein-based) offer immense advantages in biomedical applications, as they are biocompatible and biodegradable. For tissue engineering applications, protein-based biopolymers such as gelatin are preferable, as they possess an excellent loading efficiency for cells, biologics and drug delivery. Gelatin is a product of collagen hydrolysis that is native to the extracellular matrix (ECM) [12]. Gelatin is composed of heterogeneous single and multistranded polypeptides that are made up of 300–4000 amino acids [13]. Gelatin biopolymer is widely known due to its innate versatility in noncovalent interactions and phase behavior affected by pH and temperature [14]. Furthermore, its numerous functional groups (Figure 1) provide vast opportunities for chemical modification by researchers to control the biofunctional properties of gelatin [15]. Besides, gelatin biopolymer characteristically promotes cellular adhesion due to its unique amino acid sequences that facilitate cellular attachment [12].

Thus, gelatin-based microspheres (GM) provide a suitable vehicle to deliver cells and biologics, especially in the field of regenerative medicine. In this review, we attempted to describe recent works on GM, particularly in the treatment of cartilage diseases. We explored GM as an efficient drug delivery system, and its role in tissue engineering applications and cartilage regeneration.

## 2. History and Characteristics of Gelatin Microsphere

### 2.1. Early Adopters

Gelatin was famously known for its application in foods, due to its characteristic of forming a gel for food consumption. It was approved and widely used for various medical purposes, especially for drug capsules. Recent decades have seen researchers venturing into repurposing gelatin as microparticles or microspheres for controlled-delivery systems, thanks to its uniform proteolytic degradation. The original works on GM as a controlled-delivery system were begun back in the early 1960s, where Tanaka et al. first developed gelatin microspheres containing sulphanilamide or riboflavin for oral consumption, and evaluated the in vivo sustained release [16]. Following that establishment, other studies applied various processing techniques to develop GM while incorporating other therapeutic agents [17,18,19]. In the early 1990s, GM was explored for its opsonic ability for macrophage phagocytosis and antibody production in immunization [20,21]. Nowadays, numerous applications of GM in regenerative medicine are being investigated, whereby GM is used as a scaffolding material to deliver biological agents and cells for tissue regeneration.

### 2.2. Characteristics of Gelatin Microsphere

#### 2.2.1. Morphology and Size

**Figure 2:**
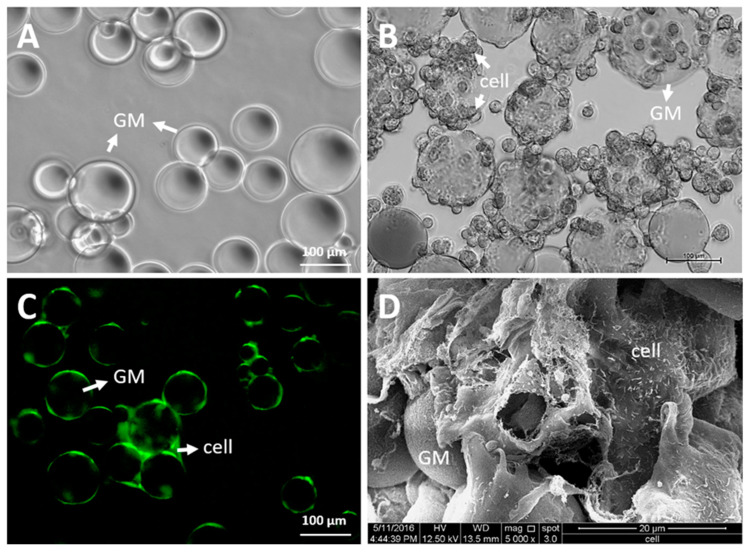
Microscopic images of gelatin microsphere (GM). (**A**) GM suspended in the buffer (under the phase-contrast microscope), (**B**) chondrocyte-laden GM (under the phase-contrast microscope), (**C**) chondrocyte-laden GM aggregates (under fluorescence microscope; green—CellTracker™ Green CMFDA fluorescent dye), and (**D**) chondrocytes attachment on GM (under scanning electron microscope). (unpublished data, Shamsul et al.).

Gelatin microspheres are spherical, and have smooth surface and sizes ranging from 6 to 200 µm in diameter [7]. The average diameter of chondrocytes is 20 µm, while the diameter of mesenchymal stem cells (MSCs) falls within the range 17–30 µm. The GMs can provide a surface for cellular attachment and proliferation, while simultaneously allowing spatial and temporal control over the release of drugs or biological agents (Figure 2). GMs of sizes between 5 and 125 µm are favorable for intra-articular administration, since this size can be monitored effortlessly through a syringe with an 18- or 20-gauge needle, while avoiding rapid clearance from the joint [22].

#### 2.2.2. Biodegradation

The biodegradability of GM can be tested through in vitro or in vivo enzymatic degradation with collagenase [21]. As is typical, an in vitro drug release study was conducted to explore the biodegradation rate of the GM. It was observed that the GM had an initial burst release in the first week and reached a plateau over the following three weeks [23,24]. Other formulations may demonstrate a cumulated release within 8 h [25]. Factors such as the amount of gelatin, the size of GM and the crosslinking degree could influence the biodegradation of GM. For instance, a higher amount of gelatin with a higher concentration of glutaraldehyde (crosslinker) would prolong the release of drugs [21,26]. Therefore, the rate of drug release is regulated by the extent of gelatin ratio and its crosslinking degree.

Its fast degradation still limits the landscape of GM use in the field of growth factor delivery. The regeneration of cartilage requires growth factors to be continuously released in situ over a more extended period (months), whereas the longest documented release of growth factor by GM was 30 days [27]. In comparison, microspheres made up of other biomaterials, such as Poly(D,L-lactic-co-glycolic acid) (PLGA)/poly (lactic acid) (PLA) and chitosan, took a longer period to be thoroughly degraded (6 weeks and 14 weeks, respectively) [28,29]. Other types of microspheres, including collagen, alginate and PCL, closely resemble the degradation profile of GM [30,31]. As previously mentioned, there is still room for optimization to tune the degradation rate of GM to suit different tissue regeneration rates.

#### 2.2.3. Biocompatibility

Gelatin, as a hydrolyzed form of collagen, is native to the ECM of tissues. Interestingly, gelatin is known to be less immunogenic than collagen, probably owing to the lack of specific amino acids, such as tyrosine, tryptophan and phenylalanine, that can trigger an immunogenic reaction [32]. Previous studies demonstrated that GM is not a cytotoxic effect of GM, thus is safe for clinical use. The study by Tan et al. showed that chondrocytes proliferated 2.3-fold after culturing for six days on a PLGA/GM scaffold [33]. Adipose stem cells remain viable at day 21 on GM [34].

#### 2.2.4. Stability

GM is reported to be stable for up to three months when stored in 75% humidity at a temperature of 37 °C [25]. The stability test was conducted to observe the ability of GM to preserve chemical or biological agents encapsulated within. Briefly, the GM was irradiated with unchanged light at 2500 LX and stored under particular conditions of humidity and temperature [25]. After storage for three months, only a slight drop in the drug was observed, and more than 90% remained after irradiation for 10 days [25].

### 2.3. Fabrication of Gelatin Microsphere

Several methods were used to prepare GM, such as coacervation phase separation [19,35], electric-field-assisted precision particle fabrication, [36] and water in oil emulsification [18,37]. Various methods of GM preparations were discussed thoroughly in a separate review article by Elzoghby (2013) [38]. Briefly, some of the techniques are reviewed below.

#### 2.3.1. Desolvation

This technique describes the addition of desolvating agent (such as alcohol or acetone) to the aqueous gelatin solution in order to dehydrate the gelatin molecules [38]. The desolvation produces large microparticles of gelatin with a wide range of sizes. The second desolvation step forms smaller and uniform GMs [39]. A crosslinking agent was added subsequently to harden the GMs. However, a modified one-step desolvation was introduced, whereby the gelatin solution is maintained at neutral pH (7.0, above isoelectric point) beforehand in order to prevent the aggregation of gelatin molecules, thus producing smaller GMs [40]. To ensure an even molecular weight distribution of gelatin, the preparation of GMs is carried out at 37 °C [40]. Overall, as described by Elzoghby, the desolvation technique has two major drawbacks, because this technique uses a toxic crosslinker and organic solvents as desolvating agents [38].

#### 2.3.2. Coacervation Phase Separation

Coacervation is a chemical process that involves the separation of two liquid–liquid phases in a suspension to produce a coacervate (rich, dense polymer) that settles down at the bottom of the solution [19,35,41]. Briefly, the gelatin solution is mixed with oil phase homogenously, and phase separation is achieved through lowering the temperature of the mix (10 °C), or by the addition of natural salts (sodium chloride/sodium sulfate) and alcohol [38]. Oppositely charged macromolecules, such as proteins or polyelectrolytes, can also be used to induce phase separation. Following phase separation, the gelatin coacervate is dehydrated, resulting in the formation of GMs.

#### 2.3.3. Electric-Field-Assisted Precision Particle Fabrication

In this technique, GM is fabricated through a series of step-wise processes that produce a constant size of microspheres. Besides, the size of the microspheres can be uniformly controlled as desired. The size is achieved by the electric-field-assisted technique that prevents the aggregation of gelatin molecules. A smooth jet of gelatin solution is generated using a dual nozzle; the inner nozzle contains a gelatin solution, while the outer nozzle contains canola oil. The gelatin solution is electrically charged, and the jet of gelatin is broken up into uniform drops by acoustic excitation. The gelatin drops are separated from one another until they gel in a cold oil bath at 0–4 °C. The resulting GMs are filtered, washed with acetone and lyophilized [36].

#### 2.3.4. Water-in-Oil Emulsification

**Figure 3:**
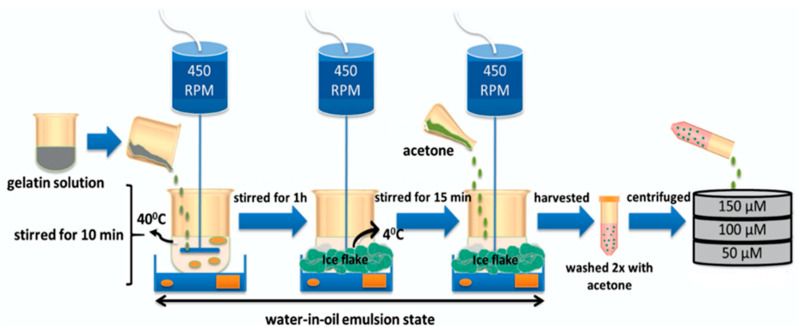
Water-in-oil emulsion method. An example of the method used to prepare gelatin microspheres.

Water-in-oil emulsification was used by most of the studies and involved several common steps (Figure 3). Gelatin powder, usually in an acidic form (isoelectric point: 4.9–5.0), is dissolved in distilled water at intended concentrations and preheated [23]. At this phase, the preparation of any drugs or biological agents is done and later mixed with the gelatin aqueous solution. For protein encapsulation by gelatin, the preheated gelatin solution is cooled down to body temperature to avoid protein denaturation, or by applying the protein solution onto the GM [42,43]. The gelatin solution is mixed with preheated oil and stirred to obtain the water in oil emulsion. The most commonly used oil is olive oil, due to its high viscosity that helps in maintaining the emulsion’s stability [42,44]. The gelling effect, intended to reverse the aqueous state of gelatin to a semi-solid state (gel), is achieved by lowering the temperature near to 0–4 °C, with constant stirring for approximately 12–30 min [45,46]. The resulting GMs are washed by acetone, and the different sizes of GMs are isolated by using sieves with variable pore sizes to filter out the GMs [47].

## 3. Gelatin Microsphere as an Intraarticular Drug Delivery System

The works of Tanaka et al., from almost 60 years ago, have since paved the way for GM to be explored as a vehicle for drug delivery in various diseases [16]. GM was previously investigated for its application as a drug-delivering agent in inflammatory bowel disease, ocular disease, and cancer therapy [18,48,49]. Several studies thus far have investigated the safety and efficacy of GM in delivering targeted drugs toward a particular site, especially in the inflammatory diseases of the joints. In terms of safety, GM was found to be non-cytotoxic in vitro and non-inflammatory in vivo [50]. This is probably due to the lesser immunogenic properties of gelatin compared with the native collagen.

In terms of efficacy, the intra-articular administration of flurbiprofen, a non-steroidal anti-inflammatory drug incorporated with GM, maintained its level in the plasma for up to 48 h, compared with the flurbiprofen solution that lasted only 12 h [25]. Meanwhile, another study has demonstrated that almost 50% of diclofenac sodium with GM can still be recovered even after a week [11]. The drug release rate depends on the degradation of the GM; different gelatin ratios/densities, the size of the microspheres and the crosslinking profile affect the biodegradability of GM [51]. However, the rate of agents released by GM could potentially be hampered in a situation where there is viscous synovial fluid, or adherence of the GM to fibrin clots, synovium and cartilage that may limit their even distribution after intra-articular administration [50].

The efficacy of GM was further elaborated by the two studies that investigated GMs loaded with drugs for the treatment of cartilage disease. Mitsui and colleagues reported that the intra-articular administration of GMs loaded with prostaglandin E2 receptor agonist (ONO-8815Ly) prevented cartilage degeneration at early stages [52]. Meanwhile, the injection of curcumin encapsulated GM–silk fibroin has demonstrated prolongued anti-inflammatory effects in osteoarthritis-induced rats [53].

## 4. Gelatin Microsphere in Cartilage Tissue Engineering Applications

The versatility of GM as an excellent controlled-delivery system has driven the particular interest among researchers in exploring its potential in the field of tissue engineering. Tissue engineering is known as a triad of stem cells, biological factors and scaffolds. Here, we attempted to elaborate on the role of GM as a scaffold or microcarrier for cells and biologic delivery, and in biologics delivery within a cartilage construct (Table 1).

### 4.1. Cells Delivery

GM was observed to be efficient in delivering cells for cartilage defects via intraarticular injection. Preliminary work on GM has shown that fabricated GMs with PLGA seeded with rabbit auricular chondrocytes facilitate chondrocyte adhesion, proliferation and viability [33]. On top of that, improved glycosoaminoglycans (GAG) secretion was seen among the GM group compared with the control group [33]. The results showed that cells grow on the surface of GMs, and therefore they are biocompatible. On the other hand, GMs could also serve as a vehicle for carrying chondrocytes within the body, to a targeted area, as shown by Leong et al. [54]. Encapsulated chondrocytes with GMs were still viable after the GMs were induced to dissolve by temperature in two days [54]. Furthermore, biochemical analyses have shown that cartilage-specific gene markers, such as collagen type-2 and glycosaminoglycans, were markedly alleviated [54].

### 4.2. Biologics Delivery

The intraarticular injection of growth factors for the treatment of cartilage diseases, such as osteoarthritis, is known for its therapeutic benefits. Biologics, such as fibroblast growth factor (FGF), platelet-rich plasma (PRP) and transforming growth factor β1 (TGF-β1), were injected to stimulate the native chondrocyte proliferation or chondrogenic differentiation of resident progenitor or stem cells. Other biologics, such as anti-inflammatory cytokines, have been utilized to suppress inflammation associated with osteoarthritis.

#### 4.2.1. Basic-Fibroblast Growth Factor

Basic-fibroblast growth factor (bFGF) is a potent mitogen that rapidly degrades upon injection or ingestion in a soluble form [55]. Thus, a transport vehicle is crucial in order to harness the proliferative effect of bFGF. In vivo studies have revealed that bFGF alone, without a controlled-release carrier, injected into osteoarthritic rabbits has led to a lesser reduction in the lesion compared with the GM-incorporated bFGF [43]. Furthermore, bFGF alone might induce an inflammatory response and osteophyte formation without significant repair when injected into an osteochondral defect at the medial femoral condyle in rabbits [56], Meanwhile, the intraarticular injections of GM-incorporated bFGF drastically suppressed the progression of osteoarthritis (OA) [43]. In the osteonecrotic OA rabbit model of the hip joint, GM and bFGF induce new bone formation, while promoting the repair of OA that improves Mankin scoring (degree of articular cartilage degeneration) [57].

#### 4.2.2. Platelet-Rich Plasma

GM was also studied for its suitability for the controlled release of PRP. Platelet-rich plasma generally consists of abundant autologous growth factors, such as platelet-derived growth factor, TGF-β and FGF, that have been used for decades for tissue regeneration. PRP was shown to maintain its biological activities, evidenced by the number of improvements seen in intervertebral disc degeneration when it was incorporated with GMs [58]. This is attributed to the sustained release of PRP from the degradation of GMs, and it therefore maintains its functionality [58]. Even in the OA model of rabbits, PRP alone without GMs resulted in the dysfunctional repair of the articular cartilage (low Mankin score) compared with the PRP-GM group [59]. This further supports that PRP can preserve its therapeutic effect when encapsulated within GMs.

#### 4.2.3. Transforming Growth Factor-Beta 1

Previous studies have discovered the potential of TGF-β1 loaded with GMs to be used to drive the chondrogenic differentiation of MSC [26,60]. Such work by Kudva et al. demonstrated the biologic delivery of TGF-β1 by GMs that stimulate the chondrogenic differentiation of human periosteum derived cells (hPDC), which are MSC-like cells. In their study, the controlled-release of TGF-β1 from GMs increased the production of GAG and collagen, the vital component of cartilage ECM, and increased the chondrogenesis of hPDC. It was also observed that the chondrogenic effect enacted by the TGF-β1 released from GMs was similar to that of the control that contains TGF-β1 within the medium [60].

#### 4.2.4. Anti-Inflammatory Cytokines

The cytokines interleukin (IL)-4, IL-10 and IL-13 have been loaded into GMs to treat osteoarthritis. It was found that IL-4 and IL-13 released from the microspheres successfully reduced chondrocyte inflammation by 65–80% within three days. GM-mediated cytokines delivery exhibited a better performance compared to the bolus treatment. Moreover, there was a significant reduction in the cytokine doses required for GM-mediated delivery to obtain a similar inflammation suppression effect [61].

### 4.3. Biologics Delivery within Cartilage Constructs

In addition to its role as a vehicle for in vivo cellular and biologics delivery, the GM was also utilized as a controlled delivery system inside an engineered tissue construct [62]. The primary purpose was to sustain the release of biologics inside the construct in a timely manner. Such an example can be portrayed by the works of Park et al., who fabricated a biodegradable polymer, oligopoly-(ethylene glycol) fumarate (OPF), a hydrogel which was then embedded with chondrocytes and GM loaded with transforming growth factor-beta 1 (TGF-beta 1). The technique produces a significant increase in chondrocyte proliferation over time [62]. A study by Fan et al. with MSCs seeded on the gelatin–chondroitin–hyaluronate scaffold with TGF-β1-loaded GM also achieved similar results in terms of cellular proliferation [63]. Surprisingly, these MSCs differentiate into the chondrocyte lineage and form ectopic cartilage when administered intraarticularly into the induced cartilage defect of mice [63]. These studies demonstrated that the controlled release of biologics is vital to ensuring success in tissue regeneration. On the other hand, exogenous TGF-β1, when added to a human adipose stem cell aggregate, only showed comparable GAG production to that of the TGF-β1-loaded GM group [45].

Several in vivo studies have assessed the efficiency of the combined gelatin–chondroitin–hyaluronate tri-copolymer scaffold and TGF-β1-loaded GMs with unprimed MSCs (GM group) in comparison with the scaffold with primed MSCs but without GMs (MSC group) [63,64]. MSCs that grew in in vitro culture with TGF-β1 added to the medium, and that differentiated into chondrocyte, were labeled “primed” MSCs. The GM group resulted in better chondrocyte morphology and tissue integration in comparison to the MSC group [63]. Moreover, they formed a thicker neocartilage layer and produced a better histological grading score than the MSC group [64]. The investigators speculated that primed MSCs were unable to retain their chondral phenotype due to the lack of in vivo cytokines from the defect itself that induce the differentiation [65]. On the contrary, the sustained release of TGF-β1 from the GMs helped in maintaining the chondral phenotype of MSCs, which subsequently resulted in better tissue remodeling and integration.

## 5. Gelatin Microsphere Effect on Chondrocyte Behavior

### 5.1. Cell Proliferation

Cartilage, a well-known tissue for limited vascularization, is populated with chondrocytes. Chondrocytes, being native cells to tissues, also contribute to the production of ECM proteins, such as collagen type-2, proteoglycan and elastin. The stimulatory effect of GM on chondrocytes’ proliferation was observed via the increased absorbance value of the MTS assay in the cell-laden GMs compared with the cell pellet alone [42]. However, the cell-laden GMs and the chondrocyte pellet failed to survive in the following 2–3 weeks of in vitro culturing [42] Conversely, the cell-laden GMs loaded with TGF-β1 were able to maintain their viability throughout the 21 days of the culture period [42]. Additionally, GMs loaded with TGF-β1 also augmented the proliferation of MSCs that grew on top of the microspheres [23]. This is partly attributed to the TGF-β1 that is prominently known to induce cellular proliferation [65].

### 5.2. Chondrogenic Differentiation

The premise of the chondrogenic effect of gelatin’s predecessor, collagen, is widely known due to its native nature as one of the building blocks for cartilage ECM. Although it is denatured collagen, gelatin retains many native collagen epitopes that support chondrogenesis. GMs alone increase the synthesis of sulfated glycosaminoglycan (sGAG), a marker of chondrogenesis, by adipose stem cells [34,35]. A recent study also indicated that GMs support chondrogenesis, as portrayed by the increased staining of the chondrogenic lineage differentiation of bone-marrow MSCs cultured on GMs [66]. It was speculated that the chondrogenic effect of GMs is attributable to the presence of amino acid sequences (arginine–glycine–aspartic acid (RGD) sequence) in gelatin protein [67].

However, most of the works that have examined the chondrogenesis effect of GMs have encapsulated growth factors within them. Such examples include the study that showed that GMs loaded with TGF-β1 significantly promoted chondrogenesis, compared to GMs alone [34]. GM-TGF-β1 also increased ECM gene expressions, such as collagen type-2 and aggrecan [68].

There was also a study that compared between the effects growth factor-loaded GMs and unloaded GMs on chondrogenesis. The study reported that MSCs cultured with unloaded GMs had lower collagen production compared with TGF-β3-loaded GMs, although not significantly. The pellets of the group with loaded GMs showed cartilage-like tissues, and were stained by alcian blue (to measure sGAG), whereas surprisingly none of these were seen in unloaded GMs. Furthermore, the unloaded GM group failed to express ECM protein and collagen types 1 and 2 [46]. It is undeniable that the growth factor plays an essential role in chondrogenesis. The unloaded GMs alone might not induce chondrogenesis without conditions that mimic the native cartilage environment. The manipulation of the culture environment that closely resembles the in vivo condition, such as by mechanical stimulation, might be able to drive cellular differentiation, as demonstrated by our recent work [66].

## 6. Future Directions

**Figure 4:**
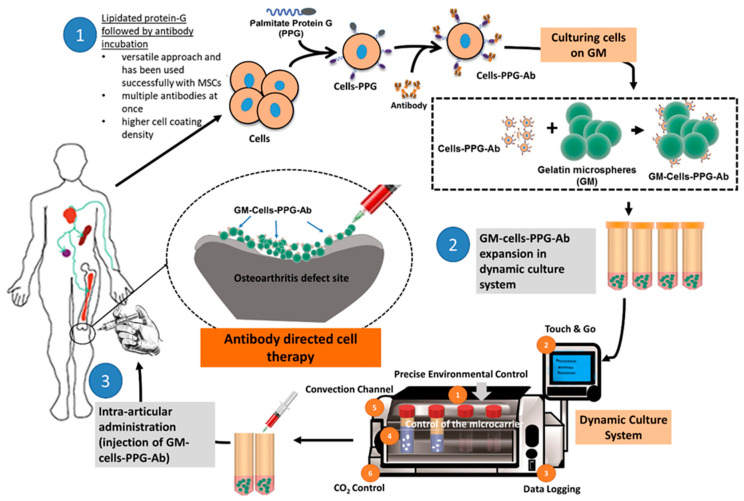
A conceptual diagram of the gelatin microsphere, together with antibody-coated cells (MSCs or chondrocytes), which can thus form an in vivo engineered tissue that will firmly bind to the injured site of the cartilage.

Despite extensive literature that has discussed the benefit of GMs in dealing with cartilage diseases, there are still specific issues that must be addressed. For instance, the action of GMs in delivering cells intraarticularly will not guarantee the attachment of the cells and the formation of neo-tissue around the injured site. Besides, the possibility of the delivered cells being suspended in the synovial fluid instead would undoubtedly raise a concern. Several studies have investigated the potential of glycoengineering in addressing the issues with targeted delivery, particularly cellular homing [69,70,71]. Conceptually, cellular homing permits the firm and strong attachment of the cells to the injured tissue. Thus cell-to-cell interactions through surface proteins and ligands could be exploited to address this. MSCs could be coated on their surface with antibodies to bind with native tissues’ cell adhesion molecules, such as intercellular cell adhesion molecule-1, thereby forming a stable cellular attachment [70]. Coating cells with antibodies is never an easy task, but is possible. Glycoengineering could indirectly coat cells with antibodies through the palmitation technique. The antibody can be linked to the cells through palmitated protein G. Protein G has a high affinity toward the binding of the Fc domain of the antibody, and can be non-covalently bonded to the cell membrane through palmitation [69]. As such, this creates an indirect antibody coating on the cell. Previously, we described the potential of GMs as an efficient delivery system for drugs, cells, and biologics. GMs have a high tendency to form aggregates in vivo; in other words, GMs could be used as the building blocks to form a large tissue construct, as shown in Figure 2D. GMs, together with antibody-coated MSCs or chondrocytes, can thus form an in vivo engineered tissue that will firmly bind to the injured site of the cartilage. Taken together, we could deliver the engineered tissue by intraarticular injection of the GMs seeded with antibody-coated cells, instead of surgical implantation, which has a higher risk of morbidity (Figure 4).

## 7. Conclusions

**Figure 5:**
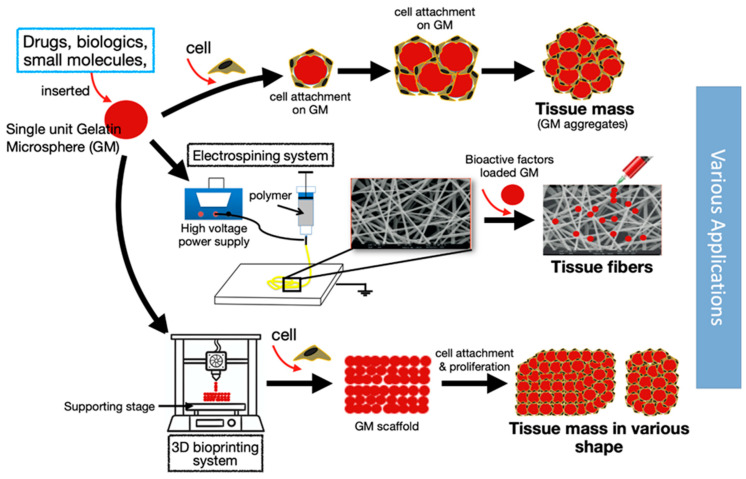
The gelatin microsphere (GM) serves as a building block in a variety of complex microsphere-based tissue constructs.

The gelatin microsphere has been investigated as an efficient drug delivery system since the 1960s, and it is only in recent decades that its potential in tissue engineering applications has been endorsed. GMs can deliver drugs and biological agents promptly, while also providing a platform for cellular delivery. In addition, GMs can potentially serve as a building block to form a more complex microsphere-based tissue construct (summarized in Figure 5). Biological agents that are important for cellular proliferation and differentiation can be encapsulated within GMs, and the proteolytic degradation of GMs by the cells on their surfaces can gradually release the “trapped” growth factors. Controlled biologics delivery is crucial for chondrogenesis due to the short half-life of most biologics—especially the growth factors, such as bFGF. The biological activity of a single injection of loaded GMs has been shown to last up to a week. GMs have also been shown to support cell differentiation, including chondrogenesis. Moreover, the possibility of targeted cell delivery and cellular homing to cartilage defects using antibody conjugation would enhance its efficacy. These techniques, when applied in a clinical setting, can potentially reduce hospital visits and costs. Taken together, the GM is beneficial as a delivery agent for both cells and biologics, and should be explored for targeted delivery for treating cartilage diseases.

## Figures and Tables

**Table 1:** Tissue engineering applications of gelatin microspheres in cartilage diseases. GM—gelatin microsphere, sGAG—sulfated glycosaminoglycan, PLGA—polylactic-glycolic acid, bFGF—basic fibroblast growth factor, ECM—extracellular matrix, OA—osteoarthritis, PRP—platelet-rich plasma, IVD—intervertebral disc, OPF—oligo (poly (ethylene glycol) fumarate, GCH—gelatin–chondroitin–hyaluronate, TGF-β1—transforming growth factor-beta 1, MSC—mesenchymal stem cell.

	References	Model	Agent	Analysis	Conclusion
Cell delivery	Tan et al. 2009 [24]	in vitro	GM + Chondrocytes	Cell viability assay Biochemical analysis (GAG)	GM with PLGA facilitated chondrocytes adhesion, proliferation, and viability. GM with PLGA improved GAG secretion.
	Leong et al. 2013 [30]	in vitro	GM + Chondrocytes	Cell viability assay sGAG/DNA analysis Gene Expression (collagen type 2, GAG) Histology IHC	GM promoted cellular proliferation. GM increased the expression of collagen type 2 and GAG.
	Cruz et al. 2013 [33]	in vitro	GM + Chondrocytes	Cell viability assay Immunofluorescence (collagen type 1 and aggrecan) Biochemical analysis (collagen type I, GAG)	GM promoted cellular proliferation.
	Xu et al. 2019 [55]	in vitro	PCL scaffold + bone marrow MSC + alginate-GM	Cell viability and proliferation sGAG/DNA analysis Gene expression Histologic analysis Mechanical test	Alginate-GM promoted cell proliferation and supported the chondrogenesis of MSC.
	Sulaiman et al. 2020 [56]	in vitro	GM + bone marrow MSC	Cell viability assay sGAG/DNA analysis Immunofluorescence (collagen type II) Gene expression Biochemical analysis (GAG)	GM increased proliferation and chondrogenesis of MSC.
	Miyakoshi et al. 2005 [47]	in vivo (osteochondral defect)	GM + bFGF	Gross morphology Histologic analysis	GM + bFGF resulted in better subchondral bone restoration (not significant).
	Inoue et al. 2006 [36]	in vivo (osteoarthritis)	GM + bFGF	ECM gene expression Gross morphology Histologic analysis	GM + bFGF reduced the progression of OA.
Biologics delivery	Nagae et al. 2007 [49]	in vivo (intervertebral disc (IVD) degeneration)	GM + PRP	Histologic analysis IHC (proteoglycan)	GM + PRP suppressed the progress of IVD degeneration.
	Saito et al. 2009 [50]	in vivo (osteoarthritis)	GM + PRP	sGAG/DNA analysis Gene expression (ECM protein) Gross morphology Histological analysis	GM + PRP increased the expression of proteoglycan (ECM protein). GM + PRP suppressed the progression of OA.
	Kuroda et al. 2010 [48]	in vivo (osteoarthritis)	GM + bFGF	Gross Morphology Histologic analysis Radiological analysis	GM + bFGF promoted repair of OA, inhibited OA progression. GM + bFGF group has lower Mankin score.
	Kudva et al. 2019 [51]	in vitro	GM + TGF-β1	sGAG/DNA analysis Biochemical analysis (collagen type I, II, GAG) Histologic analysis IHC	GM + TGF-β1 promoted chondrogenesis of human periosteum-derived cells.
	Hart et al. 2020 [52]	in vitro	GM + IL-4, IL-10, IL013	Cell viability assay Drug-released study Biochemical analysis (Nitric oxide, Nitrite)	GM loaded with interleukins (IL-4, IL-10, IL013) dramatically reduced inflammation of chondrocytes by 65-80%.
Biologics delivery in tissue scaffold	Park et al. 2005 [38]	in vitro	OPF scaffold + chondrocytes + GM+TGF-β1	sGAG/DNA analysis Histologic analysis (safranin O)	GM+ TGF-β1 increased cellular proliferation of chondrocytes.
	Fan et al. 2006 [19]	in vivo (full-thickness defect)	GCH scaffold + MSC + GM+TGF-β1	Histological analysis Gross morphology	GM+ TGF-β1 promoted tissue integration of MSC. GM+ TGF-β1 showed better chondrocyte morphology while forming new cartilage layer.
	Fan et al. 2006 [52]	in vivo	GCH scaffold + MSC + GM+TGF-β1	GAG/DNA Histological analysis of ectopic cartilage	GM+ TGF-β1 increased chondral differentiation of MSC GM+ TGF-β1 increased cellular proliferation of MSC and GAG synthesis.
	Fan et al. 2007 [53]	in vivo (full-thickness defect)	PLGA-GCH scaffold + MSC + GM+TGF-β1	Cell proliferation sGAG/DNA analysis Histological analysis Gross morphology	GM+ TGF-β1 increased cellular proliferation of MSC and GAG synthesis GM+ TGF-β1 promoted tissue integration of MSC.
	Deng et al. 2007 [60]	in vivo (full-thickness defect)	GCH scaffold + chondrocytes + GM+bFGF	Macroscopic observation Histologic analysis	GM+bFGF promoted the retention of chondrocytes and formed cartilaginous tissue in the defect.
	Yin et al. 2015 [20]	in vivo	PLGA scaffold + adipose MSC + GM+TGF-β1	sGAG/DNA analysis Histologic analysis	GM+TGF-β1 achieved better cartilage regeneration in defective articular cartilage. GM+TGF-β1 increased production of ECM protein.
